# Proportional mortality and years of potential life lost due to liver diseases among agricultural workers, Brazil, 2017 to 2022

**DOI:** 10.1590/0102-311XEN101424

**Published:** 2025-01-27

**Authors:** Jailma dos Santos Silva, Soraia Arruda, Thayane Silva Nunes, Wiler de Paula Dias, Adedayo Michael Awoniyi, Armando Meyer, Cleber Cremonese

**Affiliations:** 1 Instituto de Saúde Coletiva, Universidade Federal da Bahia, Salvador, Brasil.; 2 Hospital de Clínicas de Porto Alegre, Porto Alegre, Brasil.; 3 Instituto de Estudos em Saúde Coletiva, Universidade Federal do Rio de Janeiro, Rio de Janeiro, Brasil.

**Keywords:** Liver Diseases, Occupational Mortality, Years of Potential Life Lost, Rural Workers, Health Information Systems, Doenças do Fígado, Mortalidade Ocupacional, Anos Potenciais de Vida Perdidos, Trabalhadores Rurais, Sistemas de Informação em Saúde, Enfermedades del Hígado, Mortalidad Laboral, Años de Vida Potenciales Perdidos, Trabajadores Rurales, Sistemas de Información en Salud

## Abstract

This study aimed to describe the profile and calculate the years of potential life lost (YPLL) due to liver diseases in Brazilian agricultural workers from 2017 to 2022. For this, we analyzed microdata available in the Brazilian Mortality Information System considering the underlying cause of death with codes K70-K77 (International Classsification of Disease, 10^th^ revision − ICD-10) as the outcome of interest. Workers’ profile was characterized according to sociodemographic variables and Brazilian regions, forming a comparison group with all other Brazilian workers aged from 18-69 years who died in the same period and from the same underlying cause. Calculations of proportional mortality, YPLL rates, and YPLL rate ratios were applied. In the studied period, 15,362 deaths due to liver diseases occurred in Brazilian agricultural workers, with an average age at death of 51.3 years (±10.7), concentrated in K70 − alcoholic liver disease (53.8%). A higher proportional mortality occurred in men (86.2%), Mixed individuals (61.1%), up to age 49 years (40.9%), with ≤ 7 years of education level (52.4%), and residence in the Northeast (56.9%). The sum of YPLL totaled 382,869 years among agricultural workers, with YPLL rate of 4,527 years per 100,000 workers and a YPLL rate ratio 1.45 times higher than the national average. The concentration of deaths due to K70 raises concern due to the potential chronic exposure to alcoholic beverages. These results highlight the early causes of deaths from liver diseases among agricultural workers, especially those in Northeast Brazil and mixed.

## Resumo

O objetivo do estudo foi descrever o perfil e calcular os anos potenciais de vida perdidos (APVP) devido a doenças do fígado em trabalhadores rurais brasileiros de 2017 a 2022. Para isso, foram analisados microdados disponíveis no Sistema de Informações sobre Mortalidade (SIM), considerando o desfecho como a causa básica da morte com os códigos K70-K77 (Classificação Internacional de Doenças, 10ª revisão − CID-10). O perfil dos trabalhadores foi caracterizado de acordo com variáveis sociodemográficas e as regiões do Brasil, com um grupo de comparação formado por todos os outros trabalhadores brasileiros com idades de 18-69 anos que morreram no mesmo período e pela mesma causa básica. Foram realizados cálculos de mortalidade proporcional, assim como das taxas de APVP e suas razões. No período estudado, foram registrados 15.362 óbitos por doenças do fígado entre trabalhadores rurais brasileiros, com média de idade do óbito de 51,3 anos (±10,7), majoritariamente K70 − doença hepática alcoólica (53,8%). A mortalidade proporcional mais alta foi observada em homens (86,2%), de cor parda (61,1%), com até 49 anos (40,9%), com ≤ 7 anos de escolaridade (52,4%) e residentes do Nordeste (56,9%). A soma total de APVP foi de 382.869 anos entre trabalhadores rurais, com uma taxa de APVP de 4.527 anos por 100 mil trabalhadores e uma razão da taxa de APVP 1,45 vezes maior do que a média nacional. A concentração de mortes devido ao K70 levanta preocupação com a potencial exposição crônica a bebidas alcoólicas. Os resultados destacam as causas precoces de óbitos decorrentes de doenças do fígado entre trabalhadores rurais, especialmente aqueles da Região Nordeste do Brasil e de cor parda.

## Resumen

El objetivo de este estudio fue describir el perfil y calcular los años potenciales de vida perdidos (APVP) debido a enfermedad hepática en trabajadores rurales brasileños en el período de 2017 a 2022. Para ello, se analizaron los microdatos disponibles en el Sistema de Informaciones de Mortalidad (SIM) considerando el desenlace como la causa básica de muerte con códigos K70-K77 (Clasificación Internacional de Enfermedades, 10ª revisión − CIE-10). El perfil de los trabajadores se caracterizó con base en las variables sociodemográficas y las regiones de Brasil comparado a un grupo conformado por los demás trabajadores brasileños de entre 18 y 69 años de edad que murieron en el mismo período y por la misma causa. Se realizaron cálculos de mortalidad proporcional, así como de las tasas de APVP y sus ratios. En el período evaluado se registraron 15.362 muertes por enfermedades hepáticas entre los trabajadores rurales brasileños, con una edad media de muerte de 51,3 años (±10,7), concentrándose en K70 - enfermedad hepática alcohólica (53,8%). La mayor mortalidad proporcional se observó en hombres (86,2%), pardos (61,1%), hasta los 49 años (40,9%), con ≤ 7 años de estudios (52,4%) y residentes en el Nordeste (56,9%). La suma total de APVP fue de 382,869 años entre los trabajadores rurales, con una tasa de APVP de 4,527 años por 100.000 trabajadores y una tasa de APVP 1,45 veces más alta que el promedio nacional. La concentración de muertes por K70 evidencia una preocupación por la posible exposición crónica a bebidas alcohólicas. Los resultados indican las causas tempranas de muerte por enfermedades hepáticas entre los trabajadores rurales, especialmente los de la región Nordeste de Brasil y de color parda.

## Introduction

Liver diseases encompass a range of morbidities that impair the ability of the liver to effectively perform its functions such as substance and nutrient metabolism, blood filtration, toxin elimination, bile production, and protein synthesis ^1^. The International Classification of Diseases, 10th revision (ICD-10) ^2^ categorizes liver diseases under eight codes (K70-K77), which can result from viral infections, intoxication by harmful substances, excessive alcohol consumption, poor diet, autoimmune conditions, and genetic factors ^3^ ^,^ ^4^ ^,^ ^5^ ^,^ ^6^.

Annually, liver diseases cause 2 million deaths globally, making it the 11th leading cause of death and accounting for 4% of total mortality. Moreover, two-thirds of these deaths occur in men ^7^. Factors such as low education and non-white race/ethnicity (hereafter referred to as race) have been linked to higher incidences of liver diseases ^8^ ^,^ ^9^ ^,^ ^10^.

In low- and middle-income countries such as Brazil, factors such as sex, race, and geographic location, combined with risk behaviors and limited access to healthcare exacerbate liver dysfunction, increasing morbidity and mortality rates ^4^ ^,^ ^6^ ^,^ ^11^. The mortality rate from liver diseases showed an upward trend in Brazil from 1996 to 2012, reaching 20 deaths per 100,000 people, although with somewhat fluctuations, averaging 19 deaths per 100,000 Brazilians over the past decade. During this period, all regions showed a rising trend in these deaths, with the Northeast and Central-West regions showing a particularly higher proportion of K70 (alcoholic liver disease). Liver diseases account for 3% of all deaths in Brazil, costing the Brazilian healthcare services approximately USD 60 million per year ^12^.

Although previous studies have provided valuable insights into the epidemiological assessment of global morbidity and mortality ^5^ ^,^ ^6^ ^,^ ^11^ and liver diseases nationwide ^12^, investigations often overlook specific segments such as economic activity or type of occupation. Agricultural workers represent a distinct labor group that frequently handles pesticides and heavy metals, endures long working hours, and performs intense physical labor. Numerous studies have examined the adverse health effects of such factors on this group, focusing on chronic diseases such as neoplasms ^13^ ^,^ ^14^ ^,^ ^15^ ^,^ ^16^, cardiovascular diseases ^17^ ^,^ ^18^ ^,^ ^19^, degenerative diseases ^20^ ^,^ ^21^ ^,^ ^22^, and common mental disorders ^15^ ^,^ ^23^ ^,^ ^24^. Additionally, acute effects, including work accidents ^25^ and exogenous intoxications ^16^ ^,^ ^26^ ^,^ ^27^ are well-documented. However, studies evaluating the relationship between agricultural occupations and liver diseases, considering factors such as demographics, geographic location, occupational exposure, and access to preventive examinations or treatments remain scarce or nonexistent in Brazil.

Thus, this study aims to characterize the profile of death records from liver diseases and calculate the years of potential life lost (YPLL) among Brazilian agricultural workers in comparison to other workers, aged 18-69 years, considering sociodemographic determinants and geographical distribution across Brazil from 2017 to 2022.

## Methods

### Study design and population

This descriptive study analyzed microdata from death records due to liver diseases in individuals aged from 18-69 years across the entire Brazilian territory from 2017 to 2022 since the national and international literature has shown heterogeneity in size definition of historical series when quantifying YPLL ^28^ ^,^ ^29^ ^,^ ^30^ ^,^ ^31^ ^,^ ^32^. The final dataset includes all death records with information on the cause of death and the occupation of the deceased.

### Data collection and processing

Data were obtained from the Brazilian Mortality Information System (SIM, acronym in Portuguese), derived from death certificates provided by the Brazilian Health Informatics Department (DATASUS, acronym in Portuguese; accessible at https://datasus.saude.gov.br). To select outcomes, all records from field 40 on the death certificates (underlying cause of death) - containing codes K70 to K77 referring to liver diseases according to Chapter XI (diseases of the digestive system) of the ICD-10 were considered ^2^. Briefly, these codes include K70 (alcoholic liver disease); K71 (toxic liver disease); K72 (unspecified liver failure); K73 (chronic hepatitis); K74 (fibrosis and cirrhosis of liver); K75 (other inflammatory liver diseases); K76 (other diseases of liver); and K77 (liver disorders in diseases classified elsewhere).

The usual occupation indicated in field 14 (occupation) refers to the type of work the deceased mostly engaged in during their productive life, as per the Death Certificate Manual ^33^. The Brazilian Classification of Occupations ^34^ was used to categorize “Agricultural workers”, encompassing codes 61 (agricultural producers), 62 (agricultural workers), and 64 (agricultural and forestry mechanization workers). All other valid occupation codes were grouped under “other workers”.

Workers’ sociodemographic data were obtained from the Demographic Census available on the Automatic Recovery System of the Brazilian Institute of Geography and Statistics, specifically Table 3584, selecting the age range from 18-69 years ^35^.

To characterize the profile of deaths from liver diseases, information on sex (male, female), age groups (18-29, 30-39, 40-49, 50-59, and 60-69 years), race (white, black, yellow, mixed, and Indigenous), years of education (≤ 3, 4-7, 8-11, and ≥ 12 years), marital status (single, married, divorced, and widowed), regions (North, Northeast, Central-West, Southeast, and South), and municipality typology (urban, adjacent intermediary, remote intermediary, adjacent rural, and remote rural) ^36^ was analyzed. Missing data are shown in descriptive tables. All analyses were performed in R, version 4.1.2 (http://www.r-project.org). All datasets and codes used during this study are available in Zenodo under Creative Commons 4.0 license ^37^.

### Data analysis

The studied populations were characterized by absolute values and their corresponding frequencies. Proportional mortality (PM) was calculated by dividing the total number of deaths in the category of interest by the total number of deaths in the variable of interest for the period, multiplied by 100.

$PM=\frac{total\;number\;of\;deaths\;in\;the\;category\;of\;interest\;in\;the\;period}{total\;number\;of\;deaths\;in\;the\;variable\;of\;interest\;in\;the\;period}\times 100$ (Equation 1)

The mean age of death (MAoD) was calculated by dividing the sum of ages at death by the total number of deaths in the variable of interest.

$MAoD=\frac{sum\;of\;ages\;at\;deaths\;in\;the\;variable\;of\;interest\;in\;the\;period}{total\;number\;of\;deaths\;in\;the\;variable\;of\;interest\;in\;the\;period}$ (Equation 2)

The difference between the mean age of the variable of interest and the corresponding life expectancy (76.2 years in 2019, overall for Brazil) (mi) ^38^, multiplied by the total number of deaths in the variable of interest (ai), was considered to calculate the YPLL.

Total YPLL=∑mi×ai (Equation 3)

The percentage distribution of YPLL (%YPLL) by categories of interest was calculated by dividing the YPLL in the category of interest by the total YPLL in the variable of interest, multiplied by 100.

$\%YPLL=\frac{YPLL\;in\;the\;category\;of\;interest}{total\;YPLL\;in\;the\;variable\;of\;interest}\times 100$ (Equation 4)

The YPLL rate (YPLLr) was calculated by dividing the YPLL by the standard population, multiplied by 100,000 workers. YPLL rates were calculated by Brazilian region, agricultural workers, and other workers.

$YPLLr=\frac{YPLL\;in\;the\;category\;of\;interest}{standard\;population\;in\;the\;category\;of\;interest}\times 100,000$ (Equation 5)

Finally, the YPLLr ratios were calculated for regions and groups of workers. For regions analysis, those with the lowest YPLL rates were used as the reference (denominator) and “other workers” were used as reference (denominator) for occupation groups to facilitate the interpretation of the results.

$YPLLr\;ratio=\frac{YPLLr\;in\;the\;category\;of\;interest}{YPLLr\;in\;the\;reference\;category}$ (Equation 6)

Inferential analyses were not applied due to the census nature of the data, including all deaths registered in the investigation period, thereby eliminating the need for hypothesis testing.

### Ethical statement

The use of nonidentifiable open access data (microdata) that were available in the public domain in this study did not rEquire ethical approval.

## Results

There were a total of 4,058,039 deaths registered in SIM among adults aged 18-69 years during the study period. Of these, 120,552 cases (2.9%) showed liver diseases as the underlying cause of death (ICD-10, K70-K77). In general, MAoD totaled 52.2 years (standard deviation - SD: ±13.7); whereas for liver diseases, 53.5 years (SD: ±10.3). Deaths due to liver diseases were significantly higher among men (80.4%), those aged from 50-59 years (34.5%), those mixed (46.3%), individuals who had from eight to 11 years of education (26.4%), and single people (40.1%). Geographically, the Brazilian Southeast and Northeast and urban municipalities accounted for 43.7, 28.9, and 76.1% of these deaths, respectively (Table 1).

**Table 1:** Distribution of general deaths and those resulting from liver diseases * among adult Brazilian populations ** from 2017 to 2022.

Parameter	Deaths			
	General [n = 4,058,039]		Liver diseases [n = 120,552]	
	n	%	n	%
Sex				
Female	1,463,801	36.0	23,582	19.5
Male	2,593,835	63.9	96,957	80.4
Missing data	403	0.1	13	0.1
Mean age of death (SD)	52.2 (13.7)			53.5 (10.3)	
Age range (years)					
18-29	373,161	9.2	2,370	1.9
30-39	401,971	9.9	10,369	8.6
40-49	618,394	15.3	26,344	21.8
50-59	1,055,512	26.2	41,623	34.5
60-69	1,594,717	39.4	39,836	33.2
Race/Color				
White	1,762,347	43.4	49,260	40.8
Black	392,560	9.7	11,397	9.4
Yellow	15,687	0.4	432	0.3
Mixed	1,781,600	43.9	55,747	46.3
Indigenous	12,157	0.3	428	0.4
Missing data	93,688	2.3	3,288	2.8
Education level (years)				
≤ 3	362,897	8.9	11,698	9.7
4-7	727,277	17.9	24,905	20.6
8-11	996,511	24.6	31,748	26.4
≥ 12	993,710	24.5	25,891	21.4
Missing data	977,644	24.1	26,310	21.9
Marital status				
Single	1,540,895	37.9	48,272	40.1
Married	1,388,777	34.3	36,569	30.3
Divorced	355,700	8.8	13,790	11.4
Widowed	260,980	6.4	6,154	5.1
Missing data	511,687	12.6	15,767	13.1
Regions of Brazil				
North	295,141	7.3	6,476	5.3
Northeast	1,052,022	25.9	34,882	28.9
Central-West	302,270	7.5	9,311	7.8
Southeast	1,806,142	44.5	52,646	43.7
South	602,464	14.8	17,237	14.3
Municipal typology				
Urban	3,191,498	78.6	91,755	76.1
Adjacent intermediary	257,042	6.3	8,840	7.3
Remote intermediary	22,157	0.5	662	0.5
Adjacent rural	530,295	13.1	17,686	14.8
Remote rural	50,572	1.3	1,461	1.2
Missing data	6,475	0.2	148	0.1

SD: standard deviation.

Source: Brazilian Mortality Information System (SIM/DATASUS), and the Brazilian Ministry of Health.

* International Classification of Diseases, 10th revision (ICD-10) K70-K77 ^2^;

** 18-69 years.

Agricultural workers showed a shorter MAoD (about three years) than other workers (Table 2). Regarding PM, male agricultural workers represented 86.2% of cases, as opposed to 78.2% of other workers, whereas mixed individuals accounted for 61.1% of deaths among agricultural workers, against 43.1% among other workers. Farmers with ≤ 7 years of schooling represented 52.4%, as opposed to 27.3% from other workers. Notably, the Brazilian Northeast reported 56.9% of liver disease-related deaths among agricultural workers, which was 2.7 times higher than cases registered among other workers in the same region (Table 2).

**Table 2:** Distribution of deaths resulting from liver diseases * among agricultural and nonagricultural workers aged 18-69 years in Brazil from 2017-2022 (n = 100,190).

Parameter	Deaths resulting from liver diseases among workers			
	Agricultural ** [n = 15,362]		Others [n = 84,828]	
	n	%	n	%
Sex				
Female	2,118	13.8	18,478	21.7
Male	13,244	86.2	66,342	78.2
Missing data	-	-	8	0.1
Mean age of death (SD)	51.3 (10.7)		54.2 (10.0)	
Age range (years)				
18-29	417	2.7	1,443	1.7
30-39	1,954	12.7	6,273	7.5
40-49	3,928	25.5	17,447	20.5
50-59	5,045	32.9	29,421	34.6
60-69	4,018	26.2	30,244	35.7
Race/Color				
White	3,820	24.8	38,488	45.4
Black	1,633	10.6	7,734	9.2
Yellow	33	0.2	323	0.4
Mixed	9,400	61.1	36,638	43.1
Indigenous	168	1.2	194	0.2
Missing data	308	2.1	1,451	1.7
Education level (years)				
≤ 3	3,761	24.5	6,175	7.2
4-7	4,296	27.9	17,103	20.1
8-11	3,937	25.7	24,120	28.5
≥ 12	1,298	8.5	21,379	25.3
Missing data	2,070	13.4	16,051	18.9
Marital status				
Single	7,253	47.2	33,198	39.1
Married	4,371	28.5	27,500	32.5
Divorced	1,006	6.5	10,951	12.9
Widowed	638	4.2	4,860	5.7
Missing data	2,094	13.6	8,319	9.8
Regions of Brazil				
North	1,148	7.4	4,244	5.1
Northeast	8,742	56.9	18,173	21.4
Central-West	921	5.9	7,468	8.8
Southeast	2,948	19.3	41,502	48.9
South	1,603	10.5	13,441	15.8
Municipal typology				
Urban	5,575	36.3	71,096	83.8
Adjacent intermediary	2,402	15.6	4,830	5.7
Remote intermediary	192	1.2	361	0.4
Adjacent rural	6,605	43.0	7,864	9.3
Remote rural	586	3.4	616	0.7
Missing data	2	0.0	61	0.1

SD: standard deviation.

Source: Brazilian Mortality Information System (SIM/DATASUS), and the Brazilian Ministry of Health.

* International Classification of Diseases, 10th revision (ICD-10) K70-K77 ^2^;

** Brazilian Classification of Occupations ^34^ (CBO, 61, 62 and 64 codes).

Of the eight subdivisions of liver diseases in the ICD-10, alcoholic liver disease (K70) had the highest PM in both groups of workers. In agricultural workers, it accounted for 53.8% of deaths in this subcategory, whereas it accounted for 44.9% in other workers. The K74 subgroup, fibrosis and cirrhosis of the liver, showed the second highest PM, with 28.1% among agricultural workers and 34.3% among other workers (Table 3).

**Table 3:** Distribution of deaths resulting from liver diseases, K70-K77 subgroups of chapter XI of the International Classification of Diseases, 10th revision (ICD-10), among agricultural and nonagricultural workers aged 18-69 years in Brazil from 2017 to 2022 (n = 100,190).

ICD-10, Chapter XI - Diseases of the digestive system, K70-K77 subgroup	Deaths among workers			
	Agricultural * [n = 15,362]		Others [n = 84,828]	
	n	%	n	%
K70 - Alcoholic liver disease	8,276	53.8	38,122	44.9
K71 - Toxic liver disease	93	0.6	554	0.6
K72 - Liver failure, not elsewhere classified	1,152	7.5	6,760	7.9
K73 - Chronic hepatitis, not elsewhere classified	96	0.6	376	0.5
K74 -Fibrosis and cirrhosis of liver	4,308	28.1	28,992	34.3
K75 - Other inflammatory liver diseases	206	1.3	1,842	2.2
K76 - Other diseases of liver	1,231	8.1	8,182	9.5
K77 - Liver disorders in diseases classified elsewhere	-	-	-	-

Source: Brazilian Mortality Information System (SIM/DATASUS) and the Brazilian Ministry of Health.

* Brazilian Classification of Occupations ^34^ (CBO, 61, 62 and 64 codes); ICD-10 ^2^.

Overall, agricultural workers had lower MAoDs, except for K75 and in the Central-West. Alcoholic liver disease (K70) showed the lowest MAoD in agricultural workers with 50.1 years (SD: ±10.4), when compared to 52.8 years (SD: ±9.7) among other workers. Among agricultural workers, lower MAoDs also occurred in men (51.1 years, SD: ±10.6), black individuals (50.3 years, SD: ±10.8), mixed people (50.7 years, SD: ±10.7), Indigenous people (49.0 years, SD: ±12.1), single individuals (47.7 years, SD: ±10.6), and those in the Northeast (50.1 years, SD: ±10.9) (Table 4).

**Table 4:** Years of potential life lost (YPLL) due to liver diseases, K70-K77 subgroups of chapter XI of the International Classification of Diseases, 10th revision (ICD-10), among agricultural and nonagricultural workers * aged 18-69 years in Brazil from 2017 to 2022 (n = 15,362 agricultural; n = 84,828 others).

Variables	Mean age of death (SD)		YPLL		%YPLL	
	Agricultural	Others	Agricultural	Others	Agricultural	Others
K70-K77 ** subgroups						
K70	50.1 (10.4)	52.8 (9.7)	216,003.6	737,380.8	56.4	47.2
K71	50.7 (13.2)	52.6 (13.2)	2,371.5	10,761.6	0.6	0.6
K72	51.2 (11.7)	54.0 (11.4)	28,800.0	124,764.0	7.5	7.9
K73	51.3 (12.4)	56.0 (10.0)	2,390.4	6,100.4	0.6	0.4
K74	52.7 (10.4)	56.0 (9.3)	101,238.0	495,526.2	26.4	31.8
K75	53.2 (11.8)	52.1 (13.6)	4,738.0	38,560.0	1.3	2.5
K76	54.0 (11.1)	55.0 (10.5)	27,328.2	149,990.0	7.2	9.6
K77	-	-	-	-	-	-
Sex						
Female	52.9 (11.1)	54.7 (11.0)	49,349.4	397,277.0	13.3	21.4
Male	51.1 (10.6)	54.1 (9.8)	322,424.4	1,466,158.2	86.7	78.7
Missing data	-	53.5 (12.5)	-	181.6	-	0.9
Age range (years)						
18-29	25.8 (2.7)	25.1 (3.2)	21,016.8	73,737.3	5.5	3.9
30-39	35.4 (2.7)	35.6 (2.7)	79,723.2	254,683.8	20.8	13.7
40-49	44.8 (2.8)	45.0 (2.8)	123,339.2	544,346.4	32.6	29.2
50-59	54.4 (2.8)	54.7 (2.8)	109,981.0	632,551.5	28.6	33.9
60-69	64.2 (2.8)	64.3 (2.8)	48,216.0	359,903.6	12.5	19.3
Race/Color						
White	53.3 (10.2)	55.6 (9.5)	87,478.0	785,155.2	22.8	42.3
Black	50.3 (10.8)	53.4 (10.3)	42,294.7	176,335.2	11.1	9.5
Yellow	52.2 (10.5)	55.5 (10.6)	792.0	6,686.1	0.3	0.4
Mixed	50.7 (10.7)	53.0 (10.3)	239,700.0	850,001.6	62.7	45.7
Indigenous	49.0 (12.1)	50.0 (11.9)	4,569.6	5,082.8	1.2	0.3
Missing data	51.8 (10.7)	54.4 (10.0)	7,515.2	31,631.8	1.9	1.8
Education level (years)						
≤ 3	54.0 (9.8)	55.8 (9.8)	83,494.2	125,970.0	21.8	6.7
4-7	51.5 (10.5)	55.2 (9.5)	106,111.2	359,163.0	27.7	19.2
8-11	50.0 (10.8)	53.7 (10.0)	103,149.4	542,700.0	26.9	29.2
≥ 12	47.8 (11.5)	53.1 (10.4)	36,863.2	493,854.9	9.7	26.5
Missing data	50.7 (10.7)	54.9 (10.0)	52,785.0	341,886.3	13.9	18.4
Marital status						
Single	47.7 (10.6)	49.7 (10.5)	206,710.5	879,747.0	53.9	47.1
Married	55.6 (9.1)	57.7 (8.3)	90,042.6	508,750.0	23.6	27.3
Divorced	54.8 (8.6)	56.7 (7.8)	21,528.4	213,544.5	5.7	11.5
Widower	59.2 (8.0)	61.2 (6.6)	10,846.0	72,900.0	2.9	3.9
Missing data	50.6 (10.5)	53.2 (10.0)	53,606.4	191,337.0	13.9	10.2
Regions of Brazil						
North	52.5 (11.0)	53.2 (10.9)	27,207.6	97,612.0	7.2	5.3
Northeast	50.1 (10.9)	53.0 (10.6)	228,166.2	421,613.6	59.6	22.6
Central-West	53.5 (9.8)	52.6 (10.2)	20,906.7	176,244.8	5.4	9.4
Southeast	52.1 (10.1)	54.9 (9.7)	71,046.8	883,992.6	18.7	47.4
South	54.3 (9.5)	54.9 (9.5)	35,105.7	286,293.3	9.1	15.3
Brazil	51.3 (10.7)	54.2 (10.0)	382,513.8	1,866,216.0	-	-
Municipal typology						
Urban	52.6 (10.4)	54.5 (9.9)	131,570.0	1,542,783.2	34.7	82.9
Adjacent intermediary	51.1 (10.5)	53.1 (10.4)	60,290.2	111,573.0	15.9	5.9
Remote intermediary	48.8 (11.5)	51.2 (11.9)	5,260.8	9,025.0	1.4	0.4
Adjacent rural	51.1 (10.5)	53.0 (10.8)	165,785.5	182,444.8	43.7	9.8
Remote rural	49.7 (11.9)	51.5 (11.3)	15,529.0	15,215.2	4.1	0.9
Missing data	59.5 (0.7)	51.2 (10.3)	33.4	1,525.0	0.2	0.1

SD: standard deviation.

Source: Brazilian Mortality Information System (SIM/DATASUS), and the Brazilian Ministry of Health.

* Brazilian Classification of Occupations ^34^ (CBO, 61, 62 and 64 codes);

** K70 (alcoholic liver disease); K71 (toxic liver disease); K72 (liver failure, not elsewhere classified); K73 (chronic hepatitis, not elsewhere classified); K74 (fibrosis and cirrhosis of liver); K7 (other inflammatory liver diseases); K76 (other diseases of liver); K77 (liver disorders in diseases classified elsewhere).

During the 6-year investigation period, agricultural workers summed 382,869 YPLL, with 1,866,216 years reported among other workers. The proportional distribution of YPLL (%YPLL) by determinants showed higher proportions for both groups of workers in the K70 subgroup in men, mixed individuals, and single people. Geographical analysis showed that 59.6% of YPLL among agricultural workers significantly focused in the Northeast in comparison to only 22.6% among other workers in the same area (Table 4).

The national YPLLr for deaths associated with liver diseases totaled 3,119.9 years per 100,000 agricultural workers. Within this group, the Northeast notably had a YPLLr equal to 4,527 years per 100,000 agricultural workers. Comparing the YPLLr ratio between agricultural and other workers, that for agricultural workers was 1.44 (95% confidence interval - 95%CI: 1.37-1.53) times higher than the national average and 2.24 (95%CI: 2.12-2.36) times higher in the Northeast. Additionally, within the agricultural workers group, the Northeast showed a significantly worse situation, with a YPLLr ratio 2.78 (95%CI: 2.63-2.95) times higher than the South, which had the lowest YPLLr for liver diseases among agricultural workers (Table 5).

**Table 5:** Rate and rate ratio with 95% confidence interval (95%CI) of years of potential life lost (YPLL) resulting from liver disease * among agricultural and nonagricultural workers ** aged 18-69 years in Brazil from 2017 to 2022.

Parameter	YPLL rate ***		YPLL rate ratio		
	Agricultural	Others	Agricultural/Others (95%CI)	Agricultural/Agricultural (95%CI)	Others/Others (95%CI)
Regions of Brazil					
North	1,886.1	1,558.7	1.21 (1.13-1.29)	1.16 (1.09-1.24)	Reference (-)
Northeast	4,527.0	2,021.7	2.24 (2.12-2.36)	2.78 (2.63-2.95)	1.30 (1.21-1.39)
Central-West	2,610.8	2,563.3	1.02 (0.96-1.08)	1.61 (1.51-1.71)	1.64 (1.54-1.75)
Southeast	2,523.0	2,319.5	1.09 (1.03-1.15)	1.55 (1.46-1.65)	1.49 (1.40-1.59)
South	1,626.3	2,009.1	0.81 (0.76-0.86)	Reference (-)	1.29 (1.21-1.38)
Brazil	3,119.9	2,160.6	1.44 (1.37-1.53)	1.92 (1.81-2.04)	1.39 (1.30-1.48)

Source: Brazilian Mortality Information System (SIM/DATASUS), Brazilian Ministry of Health, and the Brazilian Institute of Geography and Statistics (IBGE).

Note: reference defined as the region with the lowest YPLL rate.

* International Classification of Diseases, 10th revision (ICD-10) K70-K77 ^2^;

** Brazilian Classification of Occupations ^34^ (CBO, 61, 62 and 64 codes);

*** YPLL rate = YPLL rate per 100,000 workers.

## Discussion

This is the first national study focusing on occupation and early deaths due to liver diseases in individuals aged from 18-69 years. Early mortality, particularly among agricultural workers, is evident from the MAoD of 51.3 years and YPLLr of 4,532.5 per 100,000 agricultural workers in the studied period. The identified type of liver disease and the concentration of deaths in Northeast Brazil, especially with higher proportions in the K70 subgroup (alcoholic liver disease) suggest explanatory hypotheses, such as possible early and excessive alcohol consumption, combined with a lack of access to preventive examinations or treatments during the early stages of liver diseases.

Diseases of the digestive system (CID-10 − Chapter XI) was the 6th leading underlying cause of death in the studied period, accounting for approximately 227,000 deaths in Brazilians aged from 18-69 years. Among these, liver diseases represented 52% of all deaths ^39^. K70-K77 pathologies offer challenges due to their multifactorial causes, including viral infections, poisoning by harmful substances, excessive alcohol consumption, high-fat diets, or genetic factors ^3^ ^,^ ^6^ ^,^ ^7^ ^,^ ^12^. Efforts in occupational health focus on understanding the direct or indirect role of work in the development of these morbidities. For example, agricultural workers are highly exposed to exogenous agents that can cause or heighten chronic diseases ^15^.

The population of agricultural workers in Brazil, the focus of this study, totals approximately 15 million individuals who are predominantly male (81%), and 45% of which identified as white and 44% as mixed, showing low education levels (70%). Economically, 70% engaged in subsistence farming, primarily producing plant-based foods and raising animals for slaughter or their derivatives (milk, wool, etc.) ^40^. These historically exploited workers still facing significant social inequalities routinely endure long working hours, exposure to various pesticides and heavy metals, vulnerability to weather conditions, and other hazards ^15^ ^,^ ^23^ ^,^ ^24^. Such conditions increase their susceptibility to work-related accidents, diseases, and health issues ^7^ ^,^ ^41^ ^,^ ^42^. Additionally, considerable distances from standard healthcare centers pose challenges for agricultural workers in accessing preventive examinations or early treatment, leading to permanent disabilities or increased risk of death.

This study created a reference group consisting of all other workers in the same age range and period for comparison. Applying no exclusion criteria included all Brazilian Classification of Occupations except agricultural workers in this group, resulting in a heterogeneous mix regarding occupational risks or protective factors for liver diseases. Notably, the reference group had twice as many white workers with higher levels of education and a geographical distribution primarily concentrated in the Southeast and in urban municipalities. Epidemiologically, these differences are important for identifying potential determinants contributing to the outcome of interest.

The use of the YPLL indicator can qualify and measure the magnitude of deaths due to specific causes of interest. In occupational health ^28^ ^,^ ^29^ ^,^ ^30^ ^,^ ^31^ ^,^ ^32^, YPLL can quantify the burden of work-related risks and, when feasible, show the determinants or exposure factors for the diseases and injuries possibly associated with deaths. YPLL calculation is recommended when the data source has good-quality dimensions, including coverage, completeness, reliability, and validity. SIM, the data source of this study, has satisfactory registration coverage for occupation among the Brazilian Health Information Systems, despite some incomplete fields ^43^. In this study, the database had an 83% completion rate for the occupation field, classified as regular in information quality ^44^. Similarly, age, which is essential to calculate YPLL, has a nearly 100% completion on the SIM records ^45^.

In this study, YPLL calculation used 76.2 years as life expectancy based on 2019 for Brazil. This research chose this year as it represents the midpoint of the studied period and the year before the onset of the COVID-19 pandemic, which eventually decreased life expectancy in subsequent years ^38^. YPLL calculations did not consider differences in sex, race, or region in which deaths occurred as it is common in national and international studies to use a single life expectancy value for YPLL calculations ^29^ ^,^ ^31^ ^,^ ^32^ ^,^ ^46^. However, it is important to note that life expectancy may vary by up to 10 years depending on regions or various other determinants. For instance, in 2019, life expectancy for men and women reached 73.1 and 80.1 years, respectively; and regionally, the Brazilian North had the lowest life expectancy (72.9 years); whereas the South, the highest (78.6 years). Similarly, the Brazilian Northeast had a 73.9-year life expectancy; the Central-West, a 75.8-year one; and the Southeast, a 78.3-year one ^38^. Historically, black and mixed individuals usually have lower life expectancy than white individuals ^31^ ^,^ ^46^ ^,^ ^47^ ^,^ ^48^, likely due to centuries of inequality and systematic disadvantages. Therefore, the potential to under- or overestimate some results must be considered. Despite this methodological limitation, the use of data analysis techniques, such as rate and rate-ratio calculations, are appropriate strategies to compare different determinants and reduce potential biases.

Men showed significantly higher incidences of all calculated indicators, both among general and agricultural workers. These findings agree with previous studies, which consistently find higher incidence and mortality rates from liver diseases in men, regardless of occupation or other determinants ^3^ ^,^ ^5^ ^,^ ^7^ ^,^ ^11^. This historical pattern of liver disease-related deaths may stem from several factors, including higher frequencies of risk behaviors such as excessive alcohol consumption and smoking, poor health-seeking behaviors, and greater environmental and occupational exposures ^4^ ^,^ ^12^ ^,^ ^23^.

The 51.3-year MAoD due to liver diseases among agricultural workers highlights the early onset of this disease in the studied population. In acute events, such as work-related accidents, it is common for the average age at death to be low, often lower than these findings. For instance, workers in the State of Bahia showed a 38.9-, 39.4-, and 40.6-year MAoD for mixed, black, and white individuals, respectively ^31^. Similarly, a study among Iranian workers found a 36-year MAoD, higher than that among Turkish workers, who had a 34-year one ^30^ ^,^ ^49^. Mortality from work-related noncommunicable diseases, such as cancers and kidney diseases, typically have higher average age at death than liver disease-related deaths ^50^ ^,^ ^51^. Premature deaths profoundly impact families disrupting the parental nucleus, losing human references and household income, and resulting in economic loss for spouses and dependents. These deaths can exacerbate social inequalities reducing the workforce of a country and negatively impacting regional and national development ^52^.

The low levels of education of agricultural workers in this study resembles findings from previous national studies using primary data ^42^ ^,^ ^53^ ^,^ ^54^ and the 2017 Agricultural Census ^40^. A high proportion of agricultural workers have seven or less years of schooling (52.4%), twice as much as other workers (27.3%), which may contribute to our main findings. When stratified by groups or regions, the low levels of education of agricultural workers show specific vulnerabilities. For example, the percentage of agricultural workers who have never attended school is higher in North and Northeast states, which may be associated with the development of liver diseases ^8^ ^,^ ^10^. This educational disparity may contribute to a diminished perception of behavioral or environmental risks in correlation with lower income and result in poor health-seeking behaviors, leading to high morbidity and mortality from liver diseases.

The lower MAoD and higher YPLL and %YPLL due to liver diseases among single individuals could stem from individuals with partners often seeking more preventive medical care than single ones, which could enable early disease detection ^55^. Another probable associated factor could refer to the high frequency of alcohol consumption among single individual agricultural workers ^56^.

Racially, the PM from liver diseases among black agricultural workers (mixed and black individuals) was 19.4% higher than that of other black workers (71.7% vs. 52.3%). Studies on workplace accidents often investigate adverse occupational effects based on race. A Brazilian study published in 2021 used SIM data and found that brown race workers had the highest average annual mortality rates due to workplace accidents nationwide ^57^. Additionally, a temporal trend analysis on fatal workplace accidents involving workers from Bahia from 2000 to 2019 concluded that mixed workers had a lower MAoD (38.9 years) and a higher average YPLL and YPLLr than black and white workers ^31^. These findings agree with the international literature, which finds that non-white and Hispanic workers have a higher prevalence of injuries, assaults, and risks related to workplace accidents ^58^ ^,^ ^59^.

Regarding the population of agricultural workers, an Epidemiological Bulletin from the Brazilian Ministry of Health using data from the Brazilian Information System for Notifiable Diseases (SINAN, acronym in Portuguese) found that 46.8% of accidents involving venomous animals and 45.4% of severe workplace accidents from 2010 to 2019 in Brazil occurred among mixed agricultural workers ^60^. Similarly, a systematic review with meta-analysis evaluating 38 international studies (United States, Australia, Belgium, Canada, China, and Finland) found higher risks of accidents and injuries among non-white agricultural workers ^61^, which is similar to the main findings of our study.

The significant proportion of YPLL (73.8%) for liver diseases among black and brown individuals entailed ignoring YPLL rates due to the limitation of lacking denominators related to occupation by race. As of May 2024, the Brazilian Institute of Geography and Statistics (IBGE, acronym in Portuguese), the main source of this data, provided no tables with this information Table 3584 ^35^. Also, tables related to occupation with information by race did not allow tabulation by specific types of occupations, as in Tables 4040 ^62^ and 3581 ^63^. Therefore, alongside determinants such as sex, age, levels of education, location, and occupation, there lies an urgent need to include race/ethnicity in information systems to enable the widespread application of health indicators for workers.

The K70 subgroup ₋ alcoholic liver disease ₋ constituted the underlying cause of death for 53.8% of the investigated agricultural workers. These findings agree with a national retrospective historical series (1996-2022) that examined various liver-related morbidities, finding alcoholic liver disease as the leading cause of death ^12^. The K70 subgroup primarily stems from chronic and high alcohol consumption ^6^ ^,^ ^7^ ^,^ ^11^. This exposure can trigger the formation of stress granules and the restructuring of hepatic tissue, degenerating various liver cells, including hepatocytes, stellate cells, cholangiocytes, and Kupffer cells ^11^. Such progressive inflammatory process replaces healthy hepatic tissue with nodules and fibrosis, compromising blood circulation ^4^.

The relationship between alcohol consumption and agricultural workers has been extensively studied ^15^ ^,^ ^20^ ^,^ ^23^ ^,^ ^24^. Potential determinants of abusive alcohol behavior include crop loss ^24^, climate change ^24^, low economic yields ^23^, psychological adversities ^23^, food insecurity ^23^, intense and exhausting workload ^23^, and high exposure to pesticides ^15^. However, scarce research has assessed the consequences of this chronic exposure and its potential adverse health outcomes, such as the development of alcoholic liver disease, which caused 8,276 deaths among Brazilian agricultural workers in the studied period.

The unfavorable outcomes for liver disease-related deaths among agricultural workers in the Brazilian Northeast support previous studies, which found adverse conditions in said workers ^29^ ^,^ ^31^ ^,^ ^41^. As about 68% of its municipalities are classified as rural, the Northeast house the largest population of subsistence agricultural workers in Brazil ^40^. Deficient infrastructure (such as basic health units, specialized centers, hospitals) and a lower number of healthcare providers, especially in remote rural areas, exacerbates early morbidity and mortality among this population ^41^. Consequently, there lies a pressing need to expand occupational health surveillance and implement measures to improve working environments and conditions. Such actions are essential to reduce the burden associated with environmental and behavioral factors such as liver diseases and promote equity among the studied population.

Reflecting on the limitations of this study, the YPLL calculation represents an absolute value, leading to higher YPLL values in large populations. To ensure comparability with studies and populations of different sizes, the YPLLr and YPLLr ratios were applied ^31^ ^,^ ^64^ as epidemiological studies commonly use these strategies ^32^ ^,^ ^46^. The YPLLr and YPLLr ratios confirmed the magnitude of the problem and highlighted the vulnerability of agricultural workers in the Brazilian Northeast. Although this research found no studies with directly comparable populations, these findings are similar to national studies, which observed premature deaths among Brazilian workers due to chronic diseases ^32^, infectious diseases ^41^, or workplace accidents ^28^ ^,^ ^31^.

Although this study relied on secondary data from SIM and IBGE, the quality of SIM records has improved significantly, particularly in the sociodemographic and occupational fields, which are now completed in 85% of the records. Additionally, the proportions of codes classified as ill-defined causes show a decreasing trend, especially within the investigated population (aged from 18-69 years).

The description of race/color on the death certificates is based on hetero-identification, inflicting a possible bias, especially the political and ideological-influenced whitening during racial identification in Brazil ^31^.

The inability to measure variables such as alcohol consumption, use of unprescribed medicine, dietary habits, and other factors limits the ability to infer causality of liver diseases among workers. Future prospective and retrospective epidemiological studies involving representative sample sizes of the worker population are necessary to better assess the potential impact of multiple risk exposures, especially on alcohol consumption and its relation to early mortality from liver diseases among agricultural workers.

Highlighting the positive aspects of this study, approximately 4 million death records formed the initial basis of its analysis. Its application of indicators such as PM and YPLL, along with YPLLr and YPLLr ratio, enabled comparisons between groups of workers based on occupational, sociodemographic, and geographic aspects, paving the way for understanding liver disease mortality among agricultural workers in Brazil. The findings of this study prompt reflection on the determinants that have rendered this group of agricultural workers particularly vulnerable to liver diseases. The concentration of deaths in the K70 subgroup − alcoholic liver disease − suggests the need for developing continuous education strategies aimed at agricultural workers to raise awareness about the risks associated with early, high, and frequent alcohol consumption. Overall, this study contributes to occupational health by fostering the development of targeted actions to promote the health and well-being of populations in agricultural activities.

## Conclusions

The 6-year investigation period in this study included 120,552 deaths associated with liver diseases in Brazilians aged from 18-69 years, of which 12.7% occurred in agricultural workers, with an MAoD of 51.3 years and a total of 382,869 YPLL. The higher proportion of deaths in the K70 subgroup suggests precocious, high, and frequent alcohol consumption among agricultural workers. The Brazilian Northeast had a YPLL rate of 4,527 years per 100,000 agricultural workers and a YPLLr ratio 1.45 times higher than the national average, rendering it the region under the most severe situation. Characterizing the vulnerability of agricultural workers to liver diseases requires combining epidemiological methods to measure behavioral, environmental, and occupational risks. This approach should help fill the knowledge gaps ignored by the scope of this study.
